# The impact on emotional well-being of being a palliative care volunteer: An interpretative phenomenological analysis

**DOI:** 10.1177/02692163211064770

**Published:** 2021-12-30

**Authors:** Helena Coleman, Andy Sanderson-Thomas, Catherine Walshe

**Affiliations:** 1 Previously of The Division of Health Research, Lancaster University, Lancaster, UK; 2 Trinity Hospice, Blackpool, UK; 3 International Observatory on End of Life Care, Lancaster University, Lancaster, UK

**Keywords:** Palliative care, terminal care, hospice care, hospices, volunteers, qualitative research

## Abstract

**Background:**

Much palliative care provision relies on the support of volunteers. Attention is paid to the risks to professionals providing care, such as stress and burnout, but understanding if this is an issue for volunteers is little understood. It is important to understand the impact their role has on volunteers emotional well-being.

**Aim:**

To explore the experiences of palliative care volunteers and how the role impacted on their emotional well-being.

**Design:**

Interpretative phenomenological analysis, with data collected through semi-structured interviews.

**Setting/participants:**

Volunteers in patient-facing roles within palliative and end-of-life care services in the UK.

**Results:**

Volunteers (*n* = 10) across three palliative and end-of-life care services. Four themes were developed: (1) it can be challenging; (2) it’s where I’m meant to be; (3) managing death; (4) the importance of connection. Challenges included frustrations and questioning themselves. Although difficult at times, volunteers expressed the importance of the role, doing well and that they benefitted too. They also had to manage death and discussed beliefs about life and death, acceptance and managing patients’ fears. Connection with the hospice, patients, staff and other volunteers was important, with a need for everyone to feel valued.

**Conclusions:**

Although there are psychosocial benefits for volunteers in their role, it is important to understand the challenges faced and consider ongoing support to help volunteers manage these challenges. This could be addressed through the consideration of coping mechanisms, further training and reflective practice for volunteers.

**What is already known about the topic?**

- Paid staff in palliative care can experience stress and burnout but little research has explored the emotional experience on volunteers.
- Volunteers provide emotional support to patients but receive little formal support.

**What this paper adds?**

- The potential impact of the challenges faced, feelings of belonging and benefitting from the role, managing death and the importance of connection with the hospice, other volunteers and patients.

**Implications for practice, theory or policy**

- The benefit of reflective practice groups to better understand how volunteers are managing within their role.
- The need for future research to focus on: gender differences and people who ceased volunteering.

## Introduction

Volunteers are an integral part of palliative care systems, with many organisations reliant on the support of volunteers for care delivery.^1^ In countries such as Germany, Switzerland and Poland, volunteers run some hospices and organisations.^2^ The USA estimates around 500,000 volunteers and the UK more than 125,000 volunteers within palliative care services.^1^ Volunteers in adult settings can be involved in many areas, including bereavement services, providing emotional support to patients and being with patients at the end of their life. Such provision of psychosocial care to patients and families^3^ could lead to increased emotional demands and psychological distress.^4^

Paid staff have typically been the focus of research investigating emotional risks including stress, burnout and mental health difficulties.^5^ It is clear that the impact of stress and burnout can be far-reaching: for example physical and mental health difficulties,^6,7^ decreased job satisfaction and leaving the profession.^8,9^ The more exposure someone has to death and dying, the higher their risk of burnout.^10^ Staff may experience compassion fatigue^11,12^ which is a secondary traumatic response experienced by those in ‘helping’ positions that are involved in the care of patients who are suffering.^13^ Training and developing individualised coping strategies to encourage self-care and resilience has been suggested to support paid staff.^14,15^ However, stress, burnout or other mental health difficulties have not been considered for volunteers in palliative care despite their regular exposure to death and dying. There is a need to consider the impact of death on volunteers’ emotional well-being and how they are coping within their role.

Volunteering across a number of settings has positive impacts for the volunteer on areas such as mental health, life satisfaction and social interaction.^16,17^ However, volunteering within palliative care can result in stressors including poor communication, lack of emotional support, feeling undervalued and the need for training.^17,18^ Research has focussed on personality characteristics of palliative care volunteers,^19^ the reasons for becoming a volunteer^20,21^ and consideration of improving and developing volunteers’ roles.^22^ This appears to be largely based on maximising the benefit of having volunteers in contrast to exploring the impact this role has on volunteers’ emotional well-being.

Palliative care volunteers can have a positive impact on patients, their families and carers; with some studies showing higher satisfaction with more volunteer involvement.^22^ Due to the reliance on volunteers, research is needed to understand the experiences of volunteering in hospice and palliative care settings.^17^ This could help to retain and recruit into these roles, whilst creating more positive experiences for volunteers.

## Method

### Research question

What are palliative care volunteers’ experiences of the impact on their emotional well-being from working in adult hospice care?

### Design

An Interpretative Phenomenological Analysis design was selected^23^ because, through interpretative methodology, it is possible to access someone’s ‘inner world’, gain a deeper understanding of a particular aspect of human experience and how individuals make meaning out of their experiences.^24,25^ A critical realist position was adopted, typical in Interpretative Phenomenological Analysis.^26^

### Setting

Three hospices in the North West of England. Hospices provide a range of services including supporting people with their physical, emotional, social and spiritual needs and can be accessed as an inpatient or outpatient.^27^ Experiences could vary greatly across settings, including the level of contact a volunteer might have with the hospice and the support they would be able to access. Experiences of hospice volunteers in patient-facing roles were selected to ensure that the sample was homogenous,^40^

### Participants

See Table 1 for inclusion and exclusion criteria.

**Table 1.** Inclusion and exclusion criteria for participants.

Inclusion criteria	Exclusion criteria
• English speaking	• Those who have been volunteering for less than one month.
• Aged 18 or over	
• Those who have the capacity to consent	
• People volunteering within palliative and end-of-life care settings with adults	
• Volunteers in patient-facing roles	

### Sampling

Interpretative Phenomenological Analysis is idiographic with an aim to understand how something has been experienced and understood.^28^ Interpretative Phenomenological Analysis research tends to have small, homogenous samples, purposively selected, and this approach was used in this study.^29^

### Recruitment

Volunteer coordinators at each participating hospice circulated information about the study to eligible volunteers by post, email or on collection. If interested, volunteers were asked to contact the researcher. For all volunteers who made contact, the lead researcher explained the study and arranged interviews. Volunteer recruitment ceased when it was determined that data collected were sufficient to address the research question.

### Data collection

Face-to-face semi-structured interviews were undertaken by HC (female researcher) in hospice and university settings. These are commonly used within Interpretative Phenomenological Analysis to elicit rich, detailed data with specific topics and flexibility for participants to discuss their own ideas.^30^ The interview schedule was generated based on previous literature and in accordance with the recommendations for Interpretative Phenomenological Analysis. Topics included becoming a volunteer, personal experiences of death and dying, the positive and challenging aspects of the role, available support, strong thoughts and feelings and how the role impacted on wider life. The participant and researcher were present. The interviews were audio-recorded and transcribed verbatim by HC. Field notes were made during and after interviews. After the first interview, data were discussed with CW and the interview schedule reviewed.

### Analysis

Interpretative Phenomenological Analysis^23^ is a double hermeneutic, so the analysis was an account of both the individuals’ meaning-making of their experience and the researcher’s interpretation of this. Transcripts were read, re-read and initial thoughts were recorded. Line-by-line analysis highlighted descriptive, linguistic and conceptual comments. Emergent themes were developed, and this process was repeated for each transcript. Patterns across cases were then documented. Participants’ individualism was maintained whilst also developing higher order concepts.^28^ Analysis was managed using NVivo12 software.^31^ Data collection ceased when sufficient high-quality data had been collected to provide a rich and detailed account of participants’ experiences. Credibility of the analysis was enhanced through discussions of transcripts, summary notes and theme descriptions between HC and CW.

### Ethical issues

Research ethics committee approval was obtained from the Faculty of Health and Medicine Research Ethics Committee (FHMREC) at Lancaster University (FHMREC reference: FHMREC18072), with research governance approval provided by participating hospice sites. Participants provided written and verbal consent. Ethical considerations included the disclosure of risk from participants and participant discomfort due to the topics. Flexibility in the interview schedule permitted the navigation of difficult topics and a plan for further support had been agreed with the hospice prior to interviewing participants. Further sources of support were highlighted to participants.

## Results

Ten hospice volunteers in patient-facing roles participated between July-October 2019. (Table 2). Both inpatient and day therapy unit roles included serving refreshments and befriending patients. Community roles were focussed on befriending and supporting with practical tasks where appropriate. The interviews lasted a mean of 84 minutes (range 63–129 minutes). Four themes were developed: (1) it can be challenging; (2) it’s where I’m meant to be; (3) managing death; (4) the importance of connection.

**Table 2.** Demographic details of participants.

Age	36–84 years old (mean 66.9 years old)
Gender	F = 6, M = 4
Ethnicity	White British *n* = 10
Marital status	Single *n* = 3
	Widow *n* = 2
	Co-habiting *n* = 1
	Married *n* = 3
	Widowed and remarried *n* = 1
Employment status	Employed full-time *n* = 1
	Employed part-time *n* = 1
	Retired *n* = 8
Length of time volunteering in current patient-facing role	9 months–17 years (mean 6.2 years, SD 6.6)
Volunteer role	Community (e.g. hospice at home, hospice neighbour) *n* = 1 (P1)
	Day therapy *n* = 2 (P8, P10)
	Inpatient unit *n* = 2 (P5, P6)
	Driver *n* = 2 (P2, P9)
	Day therapy and inpatient unit *n* = 2 (P3, P4)
	Inpatient unit and community *n* = 1 (P7)
Has a close friend or relative had EOL care in a hospice?	Yes *n* = 8
	No *n* = 2
Participants from each hospice	Hospice 1 *n* = 5, Hospice 2 *n* = 2, Hospice 3 *n* = 3
Location of interviews	Hospice = 9
	University = 1
Hospice induction and training	Hospice 1 = comprehensive induction programme including on-to-job training tailored to each role
	Hospice 2 = half day induction, on-the-job training and a series of training days for specific roles for example inpatient, befrienders and advisors
	Hospice 3 = induction programme, training for specific roles

### Theme one: It can be challenging

A commonly reported concept was difficult experiences and challenges faced when volunteering. One challenge was feeling that they were not giving enough of themselves whether that be in their skills, abilities or in time. This included feelings of not being ‘good enough’ affecting their level of confidence as they questioned if they were meeting patients’ needs. They were uncertain whether they would be able to manage other people’s experience of death and dying. This could affect their sense of worth and result in feelings of failure:

*“I just felt I never said the right thing. . .it worried me. . .I just felt inadequate” (P10, Female, <5 years volunteering).*

These challenges could lead to volunteers struggling to say ‘no’ when the hospice requested additional support, indicating the importance of boundaries. Volunteers had different boundaries; some aiming to maintain distance and not get too involved, with others wanting to maintain contact with patients after they had left the hospice:

*“It’s about retaining the little bit of distance. . .once it becomes personal. . .you’re part of that emotion so you’re not being supportive. You might need more support than that person. . .if you allow that to take over” (P7, Male, >10 years volunteering).*

Recognising limits as a volunteer was important to enable the continuation of support for patients, however sometimes this was difficult, and a break from volunteering was needed to safeguard their emotional well-being. One highlighted that the length of time with a patient could impact on emotional well-being:

*“I thought I just need to take a little breather. . .it’s very sad when anybody passes but when you’re, sometimes befriending can be a couple of weeks. . .months. . .But this was. . .nearly four and a half years”. (P7, Male, >10 years volunteering).*

Other challenges included the lack of information from the hospice regarding patients which could put volunteers in a difficult position and lead to feelings of anger or anxiety. Volunteers could be left feeling not important enough to receive particular information which could highlight a disparity between paid staff and volunteers:

*“I thought he was having a heart attack. . .his physical features told me he was having a heart attack and it wasn’t a heart attack and I should’ve been told about that. . .I was. . .a little bit annoyed with them here. . .We’ve got to know how the patient, what issues” (P9, Male, >10 years volunteering).*

Volunteers tended to only get information from the patients themselves if they chose to disclose this and some experienced this as shocking but recognised the natural human reaction of managing death:

*“You’re not human if you don’t feel it inside” (P5, Female, 5-10 years volunteering).*

Some participants described thinking about themselves as the patient which indicates that volunteering can bring death and mortality to the forefront of one’s mind.

*“I look at some of these patients and you think to yourself why them and not me? Especially if they’re young, you think how unfair is that?” (P4, Female, <5 years volunteering).*

Despite the number of challenges faced, participants described things that made them want to continue volunteering. This included faith which supported some participants in managing and making sense of challenges regarding death and accessing support from other volunteers either informally or through the hospice’s ‘buddy system’ which appeared to facilitate feelings of security within their role:

*“I think knowing that they’re there and that you’re not on your own is important. . .sometimes you think, mm am I doing it right? Am I doing it wrong?. . .it’s not something you necessarily want to take to the hospice. But you can just run it past somebody else that’s doing the same role” (P1, Female, >10 years volunteering).*

### Theme two: It’s where I’m meant to be

Volunteers talked about the hospice passionately and many expressed it being a huge part of their lives with their role providing satisfaction. Some participants lacked contentment in their day-to-day life therefore the hospice provided them with an opportunity to feel fulfilled and others discussed volunteering in the hospice as something that was meant to be and enabled self-discovery, a sense of comfort and belonging:

*“I hate my day job and I don’t have any job satisfaction. . .I get here and I know I’ve done something that’s rewarding. . .valuable, I carry that with me for the rest of the week. . .that’s my little recharge every Sunday” (P6, Female, <5 years volunteering).*

*“it was something I somehow felt drawn to. . .I just feel at home, really at home. It’s like I’ve always been there. . .Every time we went past the hospice I used to say, “one day I’m going to volunteer there”“ (P8, Female, >10 years volunteering).*

Personal benefits of volunteering were discussed including it being rewarding, a privilege and community spirited. Some felt that the hospice instilled hope and reminded them about the goodness in people and this may have helped to mitigate against the challenges:

*“This place has given me hope. . .I don’t know where else there would be such a gathering of human beings that are absolutely chuck full of kindness and humanity and sympathy and empathy and just wonderful people” (P10, Female, <5 years volunteering).*

Some participants felt that it was important to do well in their roles, for example fulfilling patients’ needs and coping with the emotions of working in an end-of-life setting. A volunteer driver found a patient dead when he arrived to pick him up. He minimised this experience. He may have felt he would not be effective in his role if he let his emotions show or it could have been a coping mechanism:

*“I think you just get immune to it. It just goes in at one ear and out at the other. Because, if you let it play on your mind you wouldn’t do it you know” (P2, Male, >10 years volunteering).*

Most participants were first exposed to the hospice through visiting a relative or friend before they became a volunteer. They discussed changing roles from visitor to volunteer, the reasons they wanted to do this and what it meant to them. Some participants wanted to maintain a connection with the hospice and be closer to their loved ones:

*“. . .when I came in, recognised the people as I say some of the nurses who were caring for my wife were still here and still are. So, I felt that was nice” (P7, Male, >10 years volunteering).*

However, some participants also discussed the hospice being different to what they had experienced as a visitor which could be difficult to accept. The nursing staff did not appear to have as much time for volunteers as they did for visitors which could leave a volunteer feeling less important, promoting a shift in their relationship:

*“That was my first kind of seeing the nurses as different people. . .I almost nearly regretted it. Coz I. . .wanted that same feeling that I had when that nurse was comforting me that day. I wanted that kind of environment all the time, just to be that whole kind of, like you’re in a big woolly blanket. And it wasn’t” (P6, Female, <5 years volunteering).*

Overall, participants had a strong desire to be part of the hospice community and reaped benefits from volunteering.

### Theme three: Managing death

Volunteers expressed how death not frightening them enabled them to effectively support patients and recognised different perceptions of death linked to faith which helped to make sense of and cope with death:

*“Some people are terribly afraid of death, they don’t want to be anywhere near it and yet it can be beautiful when people are slipping away calmly and serenely” (P10, Female, <5 years volunteering).*

Others spoke about death more frankly:

*“I blank it, it doesn’t bother me at all. . .time and time again. . .they die” (P2, Male, >10 years volunteering).*

Participants discussed their personal beliefs regarding life and death and some participants described death and illness not discriminating against people based on their status within society:

*“. . .as you know with cancer it doesn’t stop. It affects all walks of life. I had. . .the retired head of a sixth form college in the front and in the back, I had a binman. A road sweeper. . .these people went together in the hospice and they were all one really because whatever they did in their life, it didn’t matter. . .”. (P2, Male, >10 years volunteering)*

Another participant spoke about a hunger for life and being grateful for the time you have, however they highlighted that it was more challenging to accept the dying and death of younger patients:

*“. . .when it’s somebody young with a young family they’ve not really had life have they? So, in some ways they’ve been robbed of what people normally expect from life. . .Whereas somebody who’s got their grandchildren. . .It’s sad when anybody died but least, that’s life isn’t it?” (P8, Female, >10 years volunteering).*

Volunteers discussed how they support patients in their hospice journey. Participants described how they support patients with fears around death and dying and would work hard to make patients comfortable and distill these fears and perceptions around what a hospice is. However, this could put responsibility onto volunteers to share their experience of hospices within their communities:

*“It’s the word hospice that puts a lot of people off. And I just say, ‘it’s nothing like that’. I don’t come away upset or thinking about it because they’re all lovely, it’s calm, it’s a nice, calm place. There’s no screaming and carrying on” (P5, Female, 5-10 years volunteering).*

Some patients were at peace with dying but could also be fearful of death:

*“. . .sometimes it’s those last weeks that give people a certain dimension. . .A chance to make peace with their family and say goodbye and perhaps right life’s wrongs. . .this chap. . .He said, “oh I’ve said all my goodbyes” he said, “I’m ready”. . .There are some tears shed but very often there are smiles as well”(P10, Female, <5 years volunteering).*

Another participant described the importance of being alongside patients during their time in the hospice so that they did not feel alone. They also recognised the importance of giving space to the family when they visit:

*“I never used to like to think that anybody was on their own. I would even, on my break, go and have a cup of tea with someone you know just to be there. And the family would come then, and I’d just go walk away” (P5, Female, 5-10 years volunteering).*

Overall participants seemed to manage death well and had strategies to assist them. However, certain experiences were more difficult, such as seeing younger patients approaching the end of life.

### Theme four: The importance of connection

Volunteers discussed strong connections and relationships formed with patients. Volunteers spend extended periods of time with patients which can impact on the intensity of the relationship and be challenging when this ends:

*“It’s very upsetting because you’ve been with that man or that lady for sixteen weeks and then they go. . .it’s a shame when the sixteen weeks come to an end” (P4, Female, <5 years volunteering).*

In social relationships more generally, if someone is sharing personal information it can form stronger connections. Participants discussed how they connected and formed relationships with patients. Although this could be an emotional burden for volunteers, some participants spoke about the enjoyment they got from connecting with patients and meeting new people:

*“It’s a learning curve. . .you pick something up new every day. . .people with so many different tales to tell. . .I find it interesting” (P2, Male, >10 years volunteering).*

Some described reciprocity that developed in relationships with patients and families and this was important to evoke feelings of being valued by the patient:

*“When she was in the hospital she’d be saying, “I don’t want you walking down them corridors on your own”. . .she’s worrying about me. . .They get to know you and care about you as much as you hopefully care about them” (P1, Female, >10 years volunteering).*

It was important to participants to support family members as well as patients and this participant appeared to find the moment special.

It could be challenging to identify when to cease communicating with family members that volunteers have connected with after the death of their loved one. Some managed this by being guided by the family. However, the family’s needs may not reflect what is most suitable for the volunteer:

*“If they wanted to cut off dead, you know sort of when the person passes, fine. . .But if they need a little more sort of slowly releasing then I’m happy with that as well”.. (P1, Female, >10 years volunteering).*

Participants discussed the value of connection with other volunteers and the hospice. Some felt that the hospice provided them with community and fulfilment in life:

*“It just keeps me going and gets me up and gets me out” (P2, Male, >10 years volunteering).*

This indicates the value in paid staff and volunteers being treated equally and supporting volunteers to feel valued and connected to the hospice. P3 feared that this would end based on previous experiences volunteering and said:

*“That’s what I’m frightened of here. . .that you see something that you’re not keen on. . .Is that being naïve of me? That everything should be sort of sweetness and light”. (P3, Male, <5 years volunteering)*

It seemed that this participant had idealised what the hospice should be like and did not allow room for error. However, they had awareness of this which is important.

Connection played a huge part in the volunteers’ role with patients, other volunteers and the hospice. Humans seek connection and, for some volunteers who lived alone, it provided them with a community that they were not receiving elsewhere.

## Discussion

### Main findings

Being a palliative care volunteer impacted on emotional well-being. Positive impacts included feeling ‘it’s where I’m meant to be’ and ‘the importance of connection’. It enhanced the volunteers’ lives in a variety of ways. ‘Managing death’ could have a positive impact as it helped participants to understand their beliefs about life and death thus contributing to personal development, however they also had to find ways to manage patients’ fears about death. Negative impacts included challenges and frustrations that the role brought: ‘it can be challenging’.

### What this study adds

Difficult experiences were central to volunteers’ experience of their roles. Challenging experiences, which these findings support, can include seeing suffering, particularly in younger patients,^32,33^ time constraints for volunteers,^34^ not feeling confident in communicating with patients and how well volunteers’ felt they were fulfilling their role.^35^ Additionally, challenges of ‘not being good enough’, not being able to do ‘enough’ for patients and challenges regarding the hospice and its processes were identified.

Boundary challenges, such as the patient not wanting the volunteer to leave could be difficult to manage.^34^ Being able to emotionally detach from patients has been found to be an effective coping strategy for some^36^ whilst others use strategies which could be categorised as meaning-making through appraisal,^33,34,37^ problem-emotion-focussed coping.^34,37^ The current study highlights boundary challenges. It is useful to understand more about volunteers’ boundaries as it could be a challenging balance when trying to maintain positive relationships and recognising personal limits and risk of burnout. Learning how to manage emotionally was important to enable volunteers to continue in their roles. Meaning-making through appraisal was important, for example personal faith impacting on the role.^33,34,37^ Problem-focussed coping such as using internal support mechanisms (speaking with volunteer coordinators) and emotion-focussed coping (speaking to other volunteers, friends and family was also useful.^34,37^

Previous research has identified many positive impacts of being a palliative care volunteer^38^ including how the development of empathy, compassion and acceptance can positively impact the volunteers’ wider life^35,39^ as well as the development of a sense of happiness and feeling useful, thus being personally rewarding.^33,40,41^ It is also widely reported that volunteering is positively associated with better emotional well-being during older age; this could be due to a sense of accomplishment and a bigger social network therefore indicating personal gains.^42^ This research supports these understandings of the positive impact of volunteering on emotional well-being and may be helpful for volunteer coordinators in the recruitment of volunteers through promoting these rewards and benefits of volunteering.

Connection between volunteers, staff and patients were central to volunteers’ experiences. Closeness could be represented by hugging, holding hands and crying together^33^ and reciprocity within the patient-volunteer relationship is also shown.^35^ Reciprocity is an outcome of social exchange and can influence social support, physical and psychological well-being, particularly later in life.^43^ The role of a volunteer is social in nature and relationship building is important to both volunteer and patient.^44^ This research supports the importance of closeness, connection and reciprocity between patients and volunteers. The current research showed that it was important for participants to feel connection to the hospice itself but also to other volunteers as this could provide another layer of support.^36^ The personal growth experienced by participants could counteract stressful or challenging experiences within their role.^45^ It is important for hospices to understand the aspects of the volunteers’ role that are valued so highly to be able to promote this further thus impacting positively on volunteers’ emotional well-being.

### Strengths and limitations

The self-selection of participants may have meant that those who participated had stronger or more positive views and may not have been representative of the wider volunteer population. It may have been useful to also interview volunteers who had ceased volunteering as they may have found the role more emotionally challenging.

### Recommendations for future research, policy and practice

Policy makers and practitioners should consider working with volunteers to discuss helpful coping mechanisms, where they might seek professional support and channels that could be accessed to discuss patients with whom they are struggling. This could include input from the multi-disciplinary team, identify gaps in knowledge and availability of support and could be addressed from induction. Reflective practice groups, run by clinical psychologists, could be useful for volunteers to discuss patients, families, difficulties and any aspects of the therapeutic relationship.

Future research could focus on gender differences between volunteers and how they experience their roles. Research with people who decided to stop volunteering may also be useful to understand if there were any particular challenges or stressors and support hospices in recruitment and retainment of volunteers in the future. Future research is also important to further understand the rewards and benefits of volunteering

## Conclusions

This study offers insights into the impact of volunteers’ roles on their emotional well-being. Although there are psychosocial benefits for volunteers, it is also important to consider challenges and offer ongoing support to help volunteers manage these challenges.
